# Mortality Prediction in Cerebral Hemorrhage Patients Using Machine Learning Algorithms in Intensive Care Units

**DOI:** 10.3389/fneur.2020.610531

**Published:** 2021-01-20

**Authors:** Ximing Nie, Yuan Cai, Jingyi Liu, Xiran Liu, Jiahui Zhao, Zhonghua Yang, Miao Wen, Liping Liu

**Affiliations:** ^1^Department of Neurology, Beijing Tiantan Hospital, Capital Medical University, Beijing, China; ^2^China National Clinical Research Center for Neurological Diseases, Beijing, China; ^3^Department of Medicine and Therapeutics, Prince of Wales Hospital, Chinese University of Hong Kong, Hong Kong, China

**Keywords:** intracerebral hemorrhage, machine learning, mortality prediction, ICU, mimic

## Abstract

**Objectives:** This study aims to investigate whether the machine learning algorithms could provide an optimal early mortality prediction method compared with other scoring systems for patients with cerebral hemorrhage in intensive care units in clinical practice.

**Methods:** Between 2008 and 2012, from Intensive Care III (MIMIC-III) database, all cerebral hemorrhage patients monitored with the MetaVision system and admitted to intensive care units were enrolled in this study. The calibration, discrimination, and risk classification of predicted hospital mortality based on machine learning algorithms were assessed. The primary outcome was hospital mortality. Model performance was assessed with accuracy and receiver operating characteristic curve analysis.

**Results:** Of 760 cerebral hemorrhage patients enrolled from MIMIC database [mean age, 68.2 years (SD, ±15.5)], 383 (50.4%) patients died in hospital, and 377 (49.6%) patients survived. The area under the receiver operating characteristic curve (AUC) of six machine learning algorithms was 0.600 (nearest neighbors), 0.617 (decision tree), 0.655 (neural net), 0.671(AdaBoost), 0.819 (random forest), and 0.725 (gcForest). The AUC was 0.423 for Acute Physiology and Chronic Health Evaluation II score. The random forest had the highest specificity and accuracy, as well as the greatest AUC, showing the best ability to predict in-hospital mortality.

**Conclusions:** Compared with conventional scoring system and the other five machine learning algorithms in this study, random forest algorithm had better performance in predicting in-hospital mortality for cerebral hemorrhage patients in intensive care units, and thus further research should be conducted on random forest algorithm.

## Introduction

Intracerebral hemorrhage (ICH) is a common neurological emergency, accounts for ~6.5–19.6% of all strokes, and is associated with higher morbidity and mortality rates compared with ischemic strokes (1). It affects ~2 million people in the world every year (2, 3).

ICH is characterized by high mortality rate, and previous studies reported that ~35% patients die within 7 days, and 50% would die within 30 days (3, 4). The global burden of care for ICH patients is huge, especially for patients in intensive care units (ICUs). Early prediction of mortality in ICH patients is crucial for the assessment of severity of illness and adjudication of the value of novel treatments, interventions, and healthcare policies.

Several scores have been developed with the objective of predicting hospital mortality from baseline ICH patient characteristics. ICH score is one of the most commonly used scores for predicting the mortality of ICH patients (5). The score ranges from 0 to 6 and includes both clinical and radiological factors, such as Glasgow Coma Scale (GCS) score, age, infratentorial origin, ICH volume, and intraventricular hemorrhage. However, the ICH score has some limitations when used in clinical practice. The image part of ICH score needs to be assessed by experienced radiologists and neurologists; thus, it could be tricky, time consuming, and tedious for non-professional users. Acute Physiology and Chronic Health Evaluation (APACHE) II system is a widely used disease classification system in the ICU (6). It has been proven that the APACHE II can effectively predict the mortality of general ICU patients (7–10), and limited data showed that, for the ICH patients, the area under the receiver operating characteristic curve (AUC) is ~0.8 (11, 12).

Same as other clinical modules, these scores use conventional statistical analysis to identify the most relevant covariates from a set of features preselected by domain experts (13). However, in order to make it more convenient for clinical manual calculation, these models are usually simplified, which means that the weight of the model is discretized, and the number of covariates is artificially reduced, leading to the deterioration of the model performance.

By contrast, machine learning method allows the discovery of important variables and empirical patterns in data through automatic algorithms. Starting from the observation of the patient, the algorithm selects a large number of variables to identify the combination that can reliably predict the outcomes (14). With a variety of algorithms, machine learning can deal with variables with complex interactions without linear assumptions. In addition, another highlight of machine learning is that it can process a large number of predicted values, which enables the exploration of big data in a more comprehensive and in-depth way.

The current study was conducted in order to compare the results of multiple machine learning algorithms and conventional clinical scores for early prediction of mortality after ICH, based on initial clinical parameters, and attempts to optimize the model by improving algorithms.

## Materials and Methods

### Data Resources, Patient Selection, and Variables

A retrospective multicenter study was conducted using a high-quality intensive care database, Medical Information Mart for Intensive Care (MIMIC-III) (15). MIMIC-III is a large, multicenter database containing data on patients admitted to critical care units at large tertiary care hospitals, including vital signs, medications, laboratory measurements, observations and notes charted by care providers, fluid balance, procedure codes, diagnostic codes, imaging reports, hospital length of stay, survival data, etc. Part of the MIMIC-III database is extracted from the MetaVision system, and the other part is extracted from the CareVue system. The current analysis using data recorded within the first 24 h after ICU admission from the database was performed for the part extracted from the MetaVision system only so as to enhance data comparability. The study included patients aged ≥18 years and treated for ICH in the ICU during 2008–2012. The patients were identified by their ICU admission diagnosis as ICH, one of the diagnostic classifications used in the MIMIC database. Additionally, data on in-hospital mortality were obtained from the variables in the database. More details about the database can be found on the MIMIC-III website (https://mimic.physionet.org/).

### Scores

In order to determine the performance of the proposed machine learning method, APACHE II score was used as the benchmark, which provides a general measure of disease severity based on 12 conventional physiological measurements, age, and initial values of previous health conditions. APACHE II scores were collected when the database was established.

### Machine Learning Algorithms

Navicat for MySQL was used for description and visualization of MIMIC III database. A method that combines automatic algorithms and artificial selection aimed at dimension reduction was used for feature extraction from thousands of variables in this analysis. All features were selected by clinicians based on their experience in diagnosis before automatic analysis. The random forest algorithm was used for final extraction. According to the descending order of importance, the feature score higher than 0.0005 was selected for final analysis.

Multiple algorithms were chosen to improve the probability of good discrimination performance. This study used the following classifiers: nearest neighbors (NN), decision tree, neural net, AdaBoost, random forest, and gcForest, as they are the most successful and widely used models for clinical data.

### Nearest Neighbors

NN classifier classifies unlabeled observations by assigning them to the most similar labeled sample class. Both the training data set and the test data set collected the characteristics of the observation data (16). In the feature space, if most of the k nearest (i.e., the nearest) samples near a sample belonged to a certain category, then the sample would be classified to that category. When it is impossible to determine which category the current point to be classified should belong to, after taking a look at its location characteristics according to the theory of statistics and measuring the weight of its neighbors, the researchers would classify (or assign) it to the category with greater weight.

### Random Forest

Random forest algorithm is a machine learning method widely used in classification and regression, especially when the number of potential explanatory variables is far more than the observed values (17, 18). The decision tree was built by using the method in the second section, and the set of these decision trees was random forest. Each tree would get the result of classification when predicting the data, *P*_*i* = {*A*_1, *A*_2, *A*_3, …, *A*_*i* (*i* = *A*)}. The prediction results of each decision tree in I would be voted, and the one with the largest number of results would be selected as the random forest prediction value.

### AdaBoost

AdaBoost algorithm is a popular ensemble method, which combines several weak learners to improve the generalization performance (19). The mechanism is to first train a base learner from the training set and then to adjust the sample distribution according to the performance of the base learner so that more attention is paid to the previously divided samples and, at last, to train the next base learner based on the adjusted distribution. This process would be repeated until the number of learners reached the specified number, or the generalized error rate reaches certain requirements. Finally, the *T* learners were weighted and combined. The generation of the *T* + 1 learner depends on the *T* learner; thus, it is a serialization method of serial generation.

### Decision Tree

Decision tree is a kind of tree structure, in which each internal node represents a judgment on an attribute, each branch represents the output of a judgment result, and each leaf node represents a classification result (20, 21). Decision tree is a top-down, no backtracking, and continuous search for important split variables of inductive learning algorithm. Its basic goal is to construct a concise and intuitive tree structure from a group of unordered and irregular cases under the guidance of specific learning tasks.

### Training and Algorithm Optimization

The learning parameter of each algorithm was adjusted adaptively with the affinity to promote the global search ability. Accuracy and receiver operating characteristic (ROC) curve analysis were used to evaluate model identification performance. The differences were evaluated with mean AUC among the machine learning models to identify the best algorithm. The off-the-shelf methods were adopted in the Python module Scikit-Learn for the implementation of all machine learning algorithms. In order to train and validate the model, GridSearchCV method was used to optimize the performance of the model by traversing the given combination of parameters. The parameter values within the grid range were selected as the model input, and the k-fold cross-validation method was adopted to verify the accuracy of the method. The results of *K* different training groups were averaged to reduce the variance; therefore, the performance of the model would be less sensitive to the partition of data. After training on each training set, the model was tested with the corresponding test set. The final parameter values were selected for the optimal model after all steps of GridSearchCV method were completed. More details of algorithms were described in Supplementary Material.

## Results

Between 2008 and 2012, 760 ICH patients who were treated in ICU, with a mean age of 68.2 years (SD, ±15.5 years), were enrolled from MIMIC III database based on the diagnostic classifications. Among them, 583 (50.4%) patients died in hospital, and 377 (49.6%) patients survived. The median APACHE II score was 27. In the group of in-hospital death, the GCS score and APACHE II score were higher compared with the alive group. More details of baseline characteristics of the two groups are shown in Table 1.

**Table 1:** Baseline characteristics of participants.

**Characteristics**	**Overall population (*n* = 760)**	**Dead at hospital discharge (*n* = 383)**	**Alive at hospital discharge (*n* = 377)**	***P*-value**
Age, mean ± SD (years)	68.2 ± 15.5	68.0 ± 15.4	68.4 ± 15.5	0.67
Male, *n* (%)	430 (56.6)	209 (54.6)	221 (58.7)	0.26
GCS score, median (IQR)	8 (4-14)	8 (4-15)	12 (7-14)	<0.01
Eye opening	2 (1-4)	1 (1-4)	3 (1-4)	<0.01
Verbal response	1 (1-5)	1 (1–3.5)	4 (1-5)	<0.01
Motor response	5 (2-6)	4 (1-6)	6 (4-6)	<0.01
APACHE II score, median (IQR)	27 (21-33)	25 (19-32)	29 (23-34)	<0.01
Baseline systolic blood pressure, mean ± SD (mm Hg)	123.1 ± 53.7	120.5 ± 54.1	125.8 ± 53.1	0.25
Heart rate, mean ± SD (bpm)	72.1 ± 30.6	72.5 ± 30.6	71.6 ± 30.6	0.85
Respiratory rate, mean ± SD (bpm)	15.4 ± 7.1	15.7 ± 7.3	15.2 ± 7.0	0.44
Temperature, mean ± SD (F)	85.7 ± 32.6	84.9 ± 33.4	86.4 ± 31.9	0.06
WBC, mean ± SD (K/μL)	10.7 ± 5.7	10.6 ± 4.3	10.8 ± 6.8	0.31
Hematocrit, mean ± SD (%)	33.5 ± 10.0	31.0 ± 12.2	36.0 ± 6.1	<0.01
Hemoglobin, mean ± SD (mg/dL)	11.3 ± 3.5	10.5 ± 4.3	12.2 ± 2.2	<0.01
Chloride, mean ± SD (mEq/L)	98.2 ± 25.2	93.1 ± 33.2	103.4 ± 10.3	0.02
BUN, mean ± SD (mg/dL)	18.6 ± 15.2	20.1 ± 18.8	17.1 ± 10.2	0.01
Creatinine, mean ± SD (mg/dL)	1.1 ± 1.5	1.1 ± 1.9	1.0 ± 0.6	0.80
Glucose, mean ± SD (mg/dL)	138.1 ± 68.4	137.7 ± 72.6	138.4 ± 63.9	0.04
Sodium, mean ± SD (mEq/L)	131.0 ± 33.1	124.2 ± 43.8	138.0 ± 12.9	0.01
Potassium, mean ± SD (mEq/L)	3.7 ± 1.2	3.5 ± 1.4	3.9 ± 0.8	0.32
Troponin-T, mean ± SD (ng/mL)	0.1 ± 0.4	0.1 ± 0.5	0.1 ± 0.2	0.64
CK-MB, mean ± SD (ng/mL)	3.8 ± 10.8	4.3 ± 13.3	3.2 ± 7.5	0.41
CK, mean ± SD (IU/L)	242.1 ± 970.2	275.6 ± 1,180.2	208.2 ± 694.8	0.06

*APACHE, Acute Physiology and Chronic Health Evaluation; GCS, Glasgow Coma Scale; WBC, white blood cell; BUN, blood urea nitrogen; CK, creatine kinase; SD, standard deviation*.

### Feature Extraction of the Machine Learning Models

**Figure 1:**
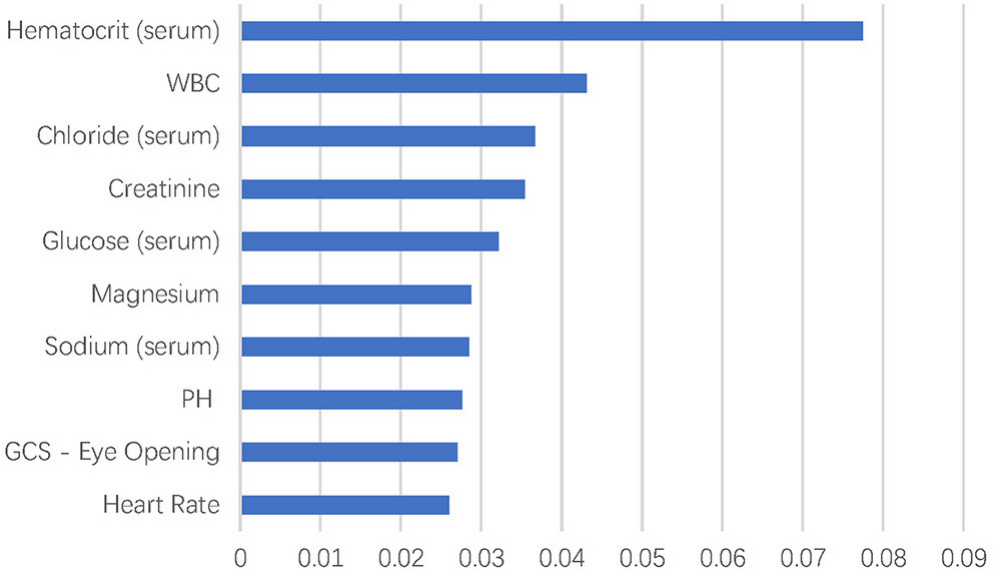
Top 10 variables in feature extraction for dimension reduction.

There were more than 10,000 variables in MIMIC III database. After selection by two ICU physicians, 2,023 variables were analyzed in feature extraction for machine learning. At last, 72 variables within the first 24 h after ICU admission were used for the training of the model. The top 10 most important variables for in-hospital mortality prediction are shown in Figure 1. The most important factor for prediction was serum hematocrit level. In addition, the top eight important variables were laboratory test results of blood biochemistry and routine blood examination of patients, including white blood cell, chloride, creatinine, glucose, magnesium, sodium, and pH. The GCS–Eye Opening score and heart rate were also affective predicting factors in the machine learning models.

### Comparison Between the Models and APACHE II Score

**Figure 2:**
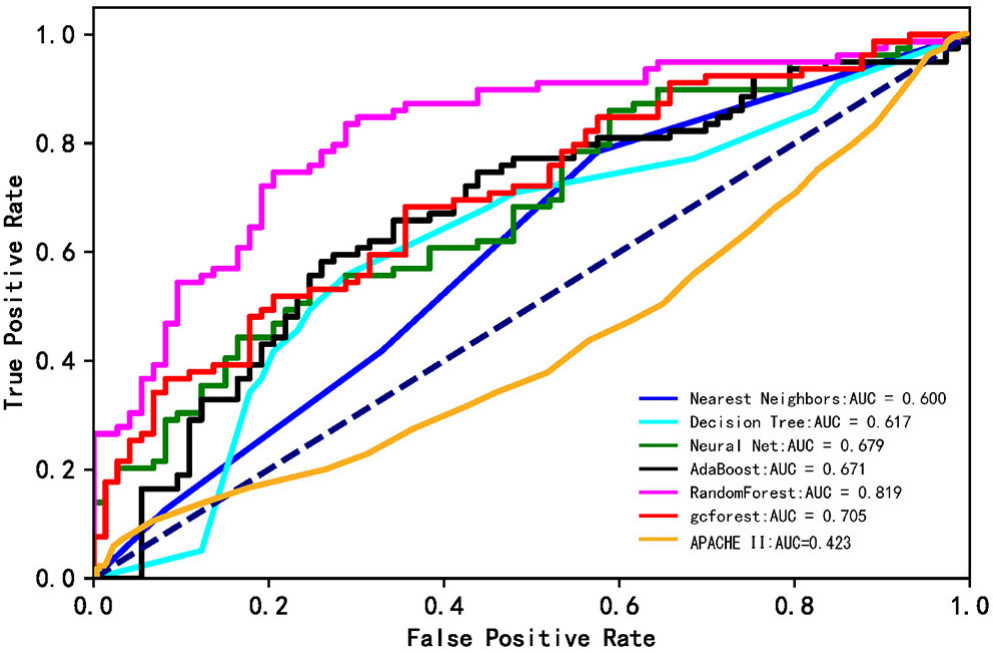
The ROC curves of the machine learning models and APACHE II score. The lines show the mean values of the scores.

Figure 2 shows ROC curves for hospital mortality prediction in external validation. The AUC for each modified model was as follows: 0.600 (nearest neighbors), 0.617 (decision tree), 0.655 (neural net), 0.671 (AdaBoost), 0.819 (random forest), and 0.725 (gcForest). However, the AUC was 0.423 for APACHE II score in the study population, which was much lower than that for machine learning models. All the machine learning algorithms showed better prediction efficiency compared with APACHE II score in the study population.

Table 2 shows the details of sensitivity, specificity, accuracy, and AUC of each model. Among the six machine learning algorithms, the random forest had the highest specificity and accuracy and the greatest AUC, showing the best ability to discriminate in-hospital mortality and survival.

**Table 2:** Comparison of sensitivity, specificity, accuracy, and AUC of each model.

**Model**	**Sensitivity**	**Specificity**	**Accuracy**	**AUC**
gcForest	0.544	0.658	0.632	0.725
Nearest neighbors	0.418	0.671	0.539	0.600
Decision tree	0.557	0.712	0.610	0.617
Neural net	0.518	0.644	0.605	0.655
AdaBoost	0.556	0.753	0.645	0.671
Random forest	0.468	0.794	0.704	0.819

*AUC, area under the receiver operating characteristic curve*.

## Discussion

The current study showed that compared with APCACHE II score, all machine learning algorithms used for prediction showed much better prediction efficiency for ICU hospital mortality in this ICH population. Among the six machine learning algorithms used in this study, the random forest was the best model for predicting the mortality of ICH patients treated in ICU. In addition, all the other five models showed moderate classification ability (ranging from 0.60 to 0.71). The current study could be considered an entirely novel exploration on the modified machine learning approach for hospital mortality prediction in ICH patients.

The result showed that all the machine learning algorithms had better ability to predict the mortality of ICH patients in this study, which may be explained by the fact that the patients in this study were treated in ICU and had severe neurological deficits. In the current study, most of the patients had conscious disturbance (median GCS, 8), and their condition is usually complicated and changes rapidly; thus, the traditional scores that are based on several preselected covariates by domain experts would be too simple to have enough power to make the correct mortality prediction (14), whereas the machine learning allows the analysis on a large number of variables simultaneously and can process the non-linear relations and the complex interactions among the variables (22). Another possible explanation would be that APACHE II is more suitable for the systemic failure patients, such as those who suffered from sepsis/septic shock (8, 9, 23), whereas for patients with abnormal vital signs and metabolic disorders caused by intracranial lesions, the variables in the scale do not have good predictive value.

In the current study, the random forest showed the best ability to discriminate in-hospital mortality and survival. The random forest had been widely used in the data analysis in neuroscience. It exhibited great ability to produce the best accuracy in many diseases and biological information analyses, such as genomic profiling, the corticospinal tract profile in amyotrophic lateral sclerosis, or the classification of neuroimaging data in Alzheimer disease (24–27). One of the merits of random forest is that it can avoid overfitting in the analysis of small sample size data (17, 18), which may explain why it had better performance in the current study. In addition, random forest has an important advantage that it has an intrinsic feature selection step applied before the classification task, which reduces the variable space by assigning an important value to each feature (28).

The model in the current study included several biochemical indicators that are considered to be related to prognosis in clinical practice but are difficult to quantify in traditional models. In the machine learning algorithm, the interaction among variables is considered in the selection of the important variables, to efficiently extract prediction patterns from data. It provides a solution different from traditional statistical screening of variables for the establishment of prediction model (17, 18). In the top 10 variables, most of the factors reflected the blood biochemistry and blood routine examination indexes, which was consistent with previous studies that showed electrolyte disorder, such as hypernatremia, is associated with clinical prognosis of cerebral hemorrhage (29). However, some of the important variables found in the current analysis were not explored in the ICH study before. Therefore, this study could also provide some insights for further hypothesis-based research.

One of the highlights of the current research is that, in the machine learning model, all the selected variables were initial clinical data and electronic monitoring data that can be automatically obtained by the monitor or can be simply evaluated (such as age, gender, GCS score), which could be automatically and dynamically assessed after simple operation by users. Although compared with the ICH scores that include radiological predictors, the prediction performance in certain aspect may be compromised, this model can be completed by nurses or assistants, thereby greatly reducing the burden of clinical work for doctors. In the future, this can even be completed by artificial intelligence monitoring instruments, achieving full automation.

To interpret the findings, it must be admitted that the current research has certain limitations. The multiple-imputation method was used to handle the missing values in the analysis, which might reduce the authenticity of the data and the accuracy of both conventional scoring system and machine learning models. Complete data will be needed in follow-up studies to improve model accuracy. The data were from the MIMIC-III database, which is a non-specialist ICU database that collects data provided by not only neurologists but also other specialists, leading to the lack of some neurologic evaluation scale data. However, its simplification is conducive to its extensive promotion among non-neurologists. In addition, the prediction model in the current study contained more variables compared with the traditional scale and showed better predictive value. In the next step, the further training of the model should be conducted with expanded sample size and improved algorithm, with the aim to further improve the prediction efficiency and reduce the required variables, so as to prepare for the next step of clinical application.

## Data Availability Statement

The datasets presented in this study can be found in online repositories. The names of the repository/repositories and accession number(s) can be found at: https://archive.physionet.org/works/MIMICIIIClinicalDatabase/files/.

## Ethics Statement

The studies involving human participants were reviewed and approved by Health Insurance Portability and Accountability Act (HIPAA) safe harbor provision. Written informed consent for participation was not required for this study in accordance with the national legislation and the institutional requirements.

## Author Contributions

LL conceived and designed the research. XN and YC acquired the data and drafted the manuscript. JL helped to analyze the data. XL and JZ helped to perform the statistical analysis. MW and ZY made critical revision of the contribution for important intellectual content. All authors contributed to the article and approved the submitted version.

## Conflict of Interest

The authors declare that the research was conducted in the absence of any commercial or financial relationships that could be construed as a potential conflict of interest.
