# Supplementary material for: Mortality Prediction in Cerebral Hemorrhage Patients Using Machine Learning Algorithms in Intensive Care Units
Source: Front Neurol. 2021 Jan 20;11:610531. doi: 10.3389/fneur.2020.610531 (PMC7855582; doi:10.3389/fneur.2020.610531)
Supplement: Supplementary file 1 [file Table_1.DOCX]

## Feature selection

A total of 2023 features were selected by clinicians based on their experience in diagnosis, and then the machine learning algorithm was used to further extract variables. The specific algorithm steps were as follows:

1. Bootstrap method was used to select n samples from the sample set as a training set

2. The sample set in step 1 was used to generate a decision tree, with each node of the decision tree satisfying the following conditions:

A. D-features were selected randomly without repetition

B. The d-features were used to divide the sample set to identify the best partition feature, and Gini coefficient was used to distinguish features

3. The variable importance measures were expressed by VIM. Gini coefficient were expressed by GIM. For the set {x1, X2, X3,..., XM} with m features, the formula (1) was used

The Gini index score of each feature X_j_ was calculated, namely, the average change of node splitting impurity of feature j in all decision trees of random forest algorithm.

$$GI_{m}=\sum_{k=1}^{\left| k \right|} \sum_{k^{’}\neq k} pmkpmk^{'}=1-\sum_{k=1}^{\left| k \right|} p^{2}mk$$

 (formula 1)

Among them, K indicates that there are k categories, and PMK represents the proportion of class k in node m, which is the probability of inconsistent class labels of two randomly selected samples from node m.

4. The importance of characteristic X_j_ in node m was calculated, and the Gini index change formula before and after node m branches was as follows

$$VIM_{j^{m}}^{\left( G_{i}n_{i} \right)}=GI_{m}-GI_{l}-GI_{r}$$

Where GI_l_ and GI_r_ are Gini indices of the two new nodes after branching respectively.

5. If the node of feature X_j_ in decision tree i was in set M, then formula (2) would be used to calculate the importance of X_j_ in the ith tree

$VIM_{\dot{l}j}^{\left( G_{ini} \right)}=\Sigma_{m\epsilon M}VIM_{j^{m}}^{\left( G_{i}n_{i} \right)}$ (formula 2)

6. If there were n trees in the random forest algorithm, then the Gini index score of each feature X_j_ would satisfy the following formula

$$VIM_{j}^{\left( G_{i}ni \right)}=\sum_{i=1}^{n} VIM_{ij}^{\left( G_{i}ni \right)}$$

7. At last, all the obtained importance scores were normalized to get the scores, using the following formula

$$VIM_{j}=\frac{VIM_{j}}{\Sigma_{i=1}^{c}VIM_{i}}$$

8. Based on the above algorithm, the importance degree of the feature could be obtained. The greater the importance degree was, the higher the influence value of the feature on the model would be. According to the descending order of the importance degree, the feature score higher than 0.0005 was selected as the final choice.

## Algorithm

**Random forest**

Same number of samples with training set were sampled randomly from the training set samples using random sampling with replacement. The number of samples in each sample set and training set was the same, but the content of each sample set was different, so as to establish different cart decision trees for training. Each tree would get the classification result, and the final result would be obtained by the voting of the trees in the forest. The algorithm flow was as follows:

For each cart decision tree, the characteristics of the sample were used as the branch node m, and the data on the M node were represented by Q. Each node had threshold value T and characteristic J, which was divided into left (q) and right (q).

$$left(Q)=(x,y)|x_{j}<=T$$

$$right(Q)=Q\backslash left(Q)$$

Where: X is the sample, y is the label, and j is the feature of sample X

The impure at branch node m was calculated recursively using Gini function

$$G\left( Q \right)=\frac{n_{\mathrm{left}}}{N}\mathrm{Gini}\left( left(Q) \right)+\frac{n_{\mathrm{right}}}{N}\mathrm{Gini}\left( \mathrm{right}(Q) \right)$$

Gini value Gini (D): two samples were randomly selected from data set D to establish a binary tree and the probability of inconsistent class labels. Therefore, the smaller Gini (D) value was, the higher the purity of data set D would be.

The formula of Gini coefficient was as follows:

$$G\mathrm{ini}\left( D \right)=\sum_{k=1}^{|y|} p_{k}(1-p_{k})=1-\sum_{k=1}^{|y|} p_{k}^{2}$$

Among them: PK is the probability of the k-class sample in the selected samples; K is the number of sample categories

When traversing each segmentation point of each feature, using feature a = a, D was divided into two parts: D1 (sample set satisfying a = a) and D2 (sample set not satisfying a = a). Then, under the condition of characteristic a = a, the formula of D would be as follows:

$$G\mathrm{ini}\left( D，A \right)=\frac{\left| D_{1} \right|}{\left| D \right|}G\mathrm{ini}\left( D_{1} \right)+\frac{\left| D_{2} \right|}{\left| D \right|}G\mathrm{ini}\left( D_{2} \right)$$

Gini (D): represents the uncertainty of set D.

Gini (A, D): represents the uncertainty of the set D divided by a = a.

Each cart decision tree in random forest was to search for the segmentation point with the smallest Gini coefficient by continuously traversing all the possible segmentation points of the feature subset of the tree. The data set was divided into two subsets until the stop condition was satisfied.

On the basis of establishing a decision tree using the above method, the set of these decision trees was random forest, and each tree would get the classification result P when forecasting data_ i={A_ 1，A_ 2，A_ 3，… ，A_ i} (I = a), P = 0_ The prediction results of each decision tree in I would be voted, and the one with the largest number of results would be selected as the random forest prediction value.

**K-nearest Neighbor Algorithm**

The basic principle of the algorithm is: in the feature space, if most of the k nearest (i.e. the nearest) samples near a sample belong to a certain category, then the sample also belongs to this category. When it’s impossible to determine which category the current point to be classified should belong to, the researchers would look at its location characteristics according to the theory of statistics, measure the weight of its neighbors, and classify it (or assign) to the category with greater weight. For each sample point, the following formula was used to calculate its distance to other I points:

$$d\left( x,y \right)=\sqrt{\sum_{i=1}^{n} {(x_{i}-y_{i})}^{2}}$$

The minimum value of K distances was selected to classify the voting. The one with the largest number of votes would be the final prediction result.

**Adaboost Algorithm**

AdaBoost is a process of upgrading a weak learner to a strong learner. The mechanism is to train a base learner from the training set, and then to adjust the sample distribution according to the performance of the base learner, so that more attention is paid to the previously misclassified samples, and then to train the next base learner based on the adjusted distribution. This process would be repeated until the number of learners reached the specified number T or the generalized error rate In the end, t learners would be weighted together. The generation of the T + 1 learner depends on the T learner, thus it is a serialization method of serial generation. The algorithm flow was as follows:

The sample set is t = {(x1, Y1), (X2, Y2),... (xn, yn)}, the initial sample weight is d (1) = (W11, W12 ,w1n)

The sample set of weight D (k) was used to train the data, and the weak classifier GK (x) was obtained, where k = 1,2,3,K;

The following formula was used to calculate the classification error rate:

$$e_{k}=\sum_{i=1}^{n} w_{ki}I\left( G_{k}\left( x_{i} \right)\neq y_{i} \right)$$

The following formula was used to calculate the weight coefficient of the weak classifier:

$$\alpha_{k}=\frac{\log\frac{1-e_{k}}{e_{k}}}{2}$$

The following formula was used to update the weight distribution of the sample set:

$$w_{k+1,i}=\frac{w_{k,i}}{Z_{k}}exp\left( -\alpha_{k}y_{i}G_{k}\left( x_{i} \right) \right)$$

Where Z_k_ is the normalization factor

$$Z_{k}=\sum_{i=1}^{n} w_{k,i}exp\left( -\alpha_{k}y_{i}G_{k}\left( x_{i} \right) \right)$$

The following formula was used to build the final classifier:

$$f\left( x \right)=sign\left( \sum_{k=1}^{k} \alpha_{k}G_{k}\left( x \right) \right)$$

## Algorithm optimization

GridSearchCV method was used to analyze these seven parameters: 'max_depth','max_features', 'min_samples_split','min_samples_leaf','bootstrap','criterion','n_estimators'.GridSearchCV is a method to optimize the performance of the model by traversing the given combination of parameters, and its full name is grid search with cross validation, namely, the combination of cross validation and grid search. With this method, the mesh range of the parameters to be optimized was set as follows:

'max_depth':[3,100],

'max_features':(1,11),

'min_samples_split': (2,11),

'min_samples_leaf': (1,11),

'bootstrap':[True,False],

'criterion':['gini','entropy'],

'n_estimators':range(10,100),

'max_depth':range(5,20)

Then a group of parameter values within the grid range were selected as the model input, and the k-fold cross validation method was used. The k value in this paper was 10. The basic idea of cross validation method is to group the original data set, and to verify the accuracy of the method with k-fold cross validation. The results of K different training groups would be averaged to reduce the variance, therefore, the performance of the model would be less sensitive to the partition of data.

In the first step, the original data were randomly divided into k copies without repeated sampling.

In the second step, one of them was selected as the test set and the remaining k-1 as the training set for model training.

In the third step, the second step was repeated for k times, so that each subset would have one chance as the test set with the rest as the training set.

After training on each training set, a model would be obtained. The model was tested with the corresponding test set. The AUC value, namely the area under the ROC curve, was calculated and the evaluation index of the model was saved. The calculation formula of AUC was as follows:

$$AUC=\frac{\sum_{i\in positiveClass} rank_{i}-\frac{M\left( 1+M \right)}{2}}{M\times N}$$

Where M is a positive sample, N is a negative sample, and rank_i_ is the probability that i test samples belongs to positive samples.

In the fourth step, the average value of AUC results of K group was calculated as the estimation of model accuracy and the performance index of the model under the current k-fold cross validation.

Finally, the above steps would be repeated to cycle through each set of optimization parameters of the grid range, until a set of optimal parameter values was selected to make the AUC index optimal.
